# Rising prevalence of systemic autoimmune rheumatic disease: increased awareness, increased disease or increased survival?

**DOI:** 10.1186/ar3954

**Published:** 2012-09-27

**Authors:** CA Peschken, CA Hitchon

**Affiliations:** 1 University of Manitoba, Winnipeg, MB, Canada

## Background

The reported prevalence of systemic autoimmune rheumatic disease (SARD) varies markedly worldwide depending on case definitions and population studied. Administrative healthcare databases provide comprehensive longitudinal datasets to estimate changing trends, but their accuracy for identifying SARD must be established, given their use for billing purposes and the diagnostic uncertainty inherent in the disease.

## Methods

In a stable population of over 900,000 adults, we used hospital and physician claims from a large administrative database using two different case definitions for the 5-year period prevalence of SARD from 1995 to 2010. For each 5-year interval, the mid-year population was used as the denominator. For physician claims, we identified SARD using The International Classification of Diseases (ICD)-9 Code 710. For hospital claims we identified SARD using the ICD-9 Code 710 from 1995 to 2004, and the ICD-10 Code M32 from 2004 onwards. Two case definitions were used: ≥5 claims ever for SARD by any physician, or ≥2 SARD claims ≥2 months apart from a specialist within the 5-year period. Prevalence rates by age (18 to 30; 31 to 50; and >50 years) and sex were calculated. The healthcare database was linked with a prescription database, and the proportion of patients treated with glucocorticoids, antimalarials and immunosuppressives was calculated.

## Results

**Figure 1:**
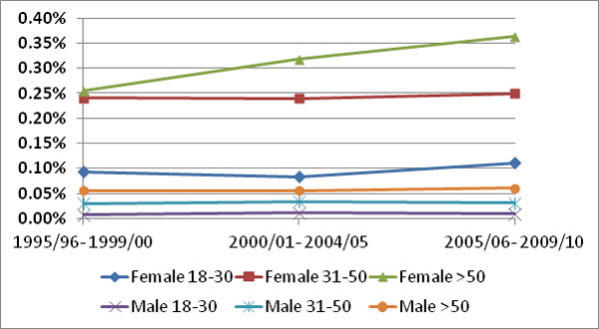
**Prevalence of SARD by age and sex**.

The prevalence rate for SARD ranged from 0.08 to 0.013% in 1995 to 2000, and rose to 0.13 to 0.16% in 2005 to 2010, depending on the case definition of SARD. Prevalence rates for females aged >50 rose from 0.25% in 1995 to 2000 to 0.36% in 2005 to 2010, while all other age and sex groups remained stable (Figure 1). The proportion of patients receiving glucocorticoid prescriptions declined slightly from 64% in 1995 to 60% in 2010, while the proportion of antimalarial, moderate and severe immunosuppressive prescriptions increased: 43 to 56%; 24 to 44%; and 3.6 to 6.6% respectively. Rheumatology manpower in the region doubled from 0.47 to 1.2/100,000 from 1995 to 2010.

## Conclusion

We found a rise in the prevalence of SARD over the 15-year period. This may reflect improved survival, given the rising rate in females >50, or improved ascertainment due to increasing awareness of SARD among physicians, and increased rheumatology manpower. This study also illustrates changing treatment patterns with less use of glucocorticoids and greater use of antimalarials and immunosuppressives, which may contribute to improved survival. Further attempts at determining diagnostic accuracy and disease outcomes in this population using linkages with clinical databases are underway.

